# Regeneration of immunocompetent B lymphopoiesis from pluripotent stem cells guided by transcription factors

**DOI:** 10.1038/s41423-021-00805-6

**Published:** 2021-12-10

**Authors:** Qi Zhang, Bingyan Wu, Qitong Weng, Fangxiao Hu, Yunqing Lin, Chengxiang Xia, Huan Peng, Yao Wang, Xiaofei Liu, Lijuan Liu, Jiapin Xiong, Yang Geng, Yalan Zhao, Mengyun Zhang, Juan Du, Jinyong Wang

**Affiliations:** 1 CAS Key Laboratory of Regenerative Biology, Guangzhou Institutes of Biomedicine and Health, Chinese Academy of Sciences, Guangzhou, 510530 China; 2 Bioland Laboratory (Guangzhou Regenerative Medicine and Health Guangdong Laboratory), Guangzhou, 510005 China; 3 Guangdong Provincial Key Laboratory of Stem Cell and Regenerative Medicine, Guangzhou Institutes of Biomedicine and Health, Chinese Academy of Sciences, Guangzhou, 510530 China; 4 University of Chinese Academy of Sciences, Beijing, 100049 China; 5 Institute for Stem Cell and Regeneration, Chinese Academy of Sciences, Beijing, 100101 China; 6 GMU-GIBH Joint School of Life Sciences, Guangzhou Medical University, Guangzhou, 511436 China

**Keywords:** Lhx2, Hoxa9, Runx1, B lymphopoiesis, pluripotent stem cells, Immunotherapy, Humoral immunity, B cells

## Abstract

Regeneration of functional B lymphopoiesis from pluripotent stem cells (PSCs) is challenging, and reliable methods have not been developed. Here, we unveiled the guiding role of three essential factors, *Lhx2, Hoxa9, and Runx1*, the simultaneous expression of which preferentially drives B lineage fate commitment and in vivo B lymphopoiesis using PSCs as a cell source. In the presence of *Lhx2, Hoxa9, and Runx1* expression, PSC-derived induced hematopoietic progenitors (iHPCs) immediately gave rise to pro/pre-B cells in recipient bone marrow, which were able to further differentiate into entire B cell lineages, including innate B-1a, B-1b, and marginal zone B cells, as well as adaptive follicular B cells. In particular, the regenerative B cells produced adaptive humoral immune responses, sustained antigen-specific antibody production, and formed immune memory in response to antigen challenges. The regenerative B cells showed natural B cell development patterns of immunoglobulin chain switching and hypermutation via cross-talk with host T follicular helper cells, which eventually formed T cell-dependent humoral responses. This study exhibits de novo evidence that B lymphopoiesis can be regenerated from PSCs via an HSC-independent approach, which provides insights into treating B cell-related deficiencies using PSCs as an unlimited cell resource.

## Introduction

B cells include invariant B-1 cells, innate-like marginal zone (MZ) B cells, and adaptive follicular (FO) B cells, all of which are essential for a functional and complete humoral immune system [1–4]. Defects in any subset of B cells can lead to severe infections from bacteria, viruses, and other microbes [5–7]. To overcome these B cell-related defects in patients, regeneration of normal B cells is an ideal approach. Researchers have attempted several approaches to produce regenerative B cells in vitro; however, it is difficult to obtain mature B cells due to the lack of methods for mimicking the spatiotemporal microenvironments of B cell development in the natural spleen. In the presence of MS5 stromal cells, CD34^+^ blood progenitors can differentiate into B cell precursors and IgM^+^ B cells in vitro [8, 9]. CD93^+^ B progenitor cells and functional IgM^+^ B lymphocytes can be generated in vitro from a mouse embryonic stem cell (ESC)/OP9 coculture system with the addition of exogenous Flt-3L [10]. Human iPSCs cocultured with stromal cells in vitro are able to differentiate into IgM^+^ B cells [11]. Mouse ESC-derived pro/pre-B cells can transiently produce B-1 and conventional B cells in Rag-deficient mice [12]. More recently, a study demonstrated that ESC-derived B progenitors induced long-term production of B-1b and MZ B cells but failed to produce FO B cells in vivo [13]. Similarly, incomplete B cell populations were generated in recipients transplanted with ESC-derived c-Kit^+^ hematopoietic progenitors [14]. Of note, a conventional strategy to regenerate engraftable B lymphopoiesis from PSCs is to produce HSC-like cell intermediates with complete blood lineage potential [15, 16]. However, generating engraftable HSC-like cells in vitro is extremely inefficient [17]. Nonetheless, an efficient approach for regenerating entire subsets of functional B-1 and B-2 cells from PSCs, either in vitro or in vivo, has not been successfully developed.

Recent studies have shown that yolk sac (YS) and para-aortic splanchnopleura (P-Sp) cells can generate B-1 progenitors [18, 19]. Pre-HSCs isolated from the YS and P-Sp are also capable of producing B-1 and B-2 cells [20, 21], indicating that the B-1 and B-2 cell fates are determined before the emergence of definitive HSCs. Our group recently reported that induced hemogenic endothelial progenitors (iHECs) derived from embryonic stem cells with inducible expression of *Runx1* and *Hoxa9* can generate induced hematopoietic progenitor cells (iHPCs) that preferentially contribute to the production of functional T cells in vivo [22]. Thus, regeneration of lymphopoiesis from PSCs can be achieved in the absence of regenerative HSCs.

In this study, we identified that synergistic expression of *Lhx2*, *Hoxa9*, and *Runx1* dominantly confers a B cell lineage fate on PSC-derived iHPCs and leads to complete B lymphopoiesis in vivo following a differentiation scheme we previously reported [22, 23]. The regenerative B (iB) cells, including B-1a, B-1b, FO B, and MZ B cell subsets, possess diverse BCR repertoires similar to their natural B cell counterparts. These iB cells can restore antibody responses triggered by specific antigen inoculation and maintain long-term humoral protection in B cell-deficient mouse. For the first time, in the absence of iHSCs, we established a de novo approach for exclusively generating functional and complete B lymphopoiesis using ESC-derived iHPCs, which provides insights into regenerative B cell therapy.

## Materials and methods

### Mice

μMT (B6.129S2-Ighmtm1Cgn/J, CD45.2^+^) mice were purchased from The Jackson Laboratory. C57BL/6 (CD45.2^+^) mice were purchased from Beijing Vital River Laboratory Animal Technology. *Rag1*^−/−^ mice (C57BL/6 background, CD45.1^+^) were a gift from Dr. Zhihua Liu of the Institute of Biophysics (CAS, China). Mice were housed in the SPF-grade animal facility of the Guangzhou Institutes of Biomedicine and Health, Chinese Academy of Sciences (GIBH, CAS, China). All animal experiments were approved by the Institutional Animal Care and Use Committee of Guangzhou Institutes of Biomedicine and Health (IACUC-GIBH).

### Gene editing

To generate GFP-reporter ESCs (GFP-ESCs), the *CAG Pr-GFP-PGK Pr-PuroR* cassette was inserted into the *Hipp11* locus of mouse ESCs (C57BL/6 background, CD45.2 strain) by homologous recombination. The positive clones (GFP-ESCs) were selected by puromycin (1 μg/mL, Thermo Fisher Scientific), and the expression of GFP was confirmed by flow cytometry. To generate *iRunx1-Hoxa9-Lhx2* (*iR9X2*) ESCs, a *CAG Pr-rtTA-3×Stop-TRE-Runx1-p2a-Hoxa9-t2a-Lhx2-pA-PGK Pr-HygroR* cassette was inserted into the *Rosa26* locus of GFP-ESCs by homologous recombination. The positive clones (*iR9X2*-ESCs) selected by hygromycin B (150 μg/mL, InvivoGen) were further cultured in ES medium supplemented with doxycycline (1 μg/mL, Sigma), and the induced expression of *Runx1*, *Hoxa9*, and *Lhx2* was confirmed by qPCR. A *CAG Pr-rtTA-3×Stop-TRE-Runx1-p2a-Lhx2-PGK Pr-HygroR* cassette was inserted into the *Rosa26* locus of GFP-ESCs by homologous recombination to generate *iRunx1- Lhx2* ESCs. Positive clones (*iRunx1-Lhx2*-ESCs) selected by hygromycin B (150 μg/mL, InvivoGen) were further cultured in ES medium supplemented with doxycycline (1 μg/mL, Sigma), and the induced expression of *Runx1* and *Lhx2* was confirmed by qPCR. To generate GFP-negative *iRunx1-Hoxa9-Lhx2-*ESCs, a *CAG Pr-rtTA-3×Stop-TRE-Runx-p2a-Hoxa9-t2a-Lhx2-pA-PGK Pr-HygroR* cassette was inserted into the *Rosa26* locus of mouse ESCs (C57BL/6 background, CD45.2 strain) by homologous recombination. The positive clones selected by hygromycin B (150 μg/mL, InvivoGen) were further cultured in ES medium supplemented with doxycycline (1 μg/mL, Sigma), and the induced expression of *Runx1*, *Hoxa9*, and *Lhx2* was confirmed by qPCR.

### Cell culture

Mouse embryonic fibroblasts (MEFs) were derived from 13.5 d.p.c. C57BL/6 mouse embryos. MEFs were maintained in DMEM/high glucose (HyClone) and 10% FBS (Natocor) supplemented with 1% nonessential amino acids (NEAAs, Gibco). C57BL/6 mouse embryonic stem cells (Biocytogen), including GFP-ESCs, *iRunx1-Hoxa9-Lhx2-*ESCs, and *iRunx1-Lhx2-*ESCs, were maintained on feeder layers in ES medium containing DMEM/high glucose, 15% FBS (Gibco), 1% NEAA (Gibco), 1% GlutaMAX (Gibco), 1% sodium pyruvate (Gibco), 0.1 mM β-mercaptoethanol (Sigma), 1 μM PD0325901 (Selleck), 3 μM CHIR-99021 (Selleck), and 1000 U/mL LIF (PeproTech). OP9-DL1 cells (GFP^+^) were maintained in α-MEM (Gibco) supplemented with 20% FBS (Ausbian). The AFT024 cell line (ATCC) was maintained in DMEM/high glucose and 10% FBS (Natocor) supplemented with 0.1 mM β-mercaptoethanol and 1% sodium pyruvate.

### Hematopoietic differentiation

ESCs were trypsinized with 0.05% trypsin-EDTA (Gibco) and resuspended in basic differentiation medium (BDM: IMDM, 15% FBS (Gibco), 200 μg/mL iron-saturated transferrin (Sigma), 0.1 mM β-mercaptoethanol (Sigma), 1% GlutaMAX, and 50 μg/mL ascorbic acid (Sigma)). To remove the feeder layers, the PSCs were plated into 0.1% gelatin-coated (Merck Millipore) wells, and the floating cells were collected after 30 mins. For embryoid body (EB) generation, the PSCs were resuspended at 100,000 cells/mL in BDM supplemented with 5 ng/mL BMP4 (Peprotech) and plated at 20 μL/drop for inverted culture in 15 cm dishes. On Day 2.5, EBs were replanted into gelatinized plates in BDM supplemented with 5 ng/mL BMP4 and 5 ng/mL VEGF (Novoprotein). On Day 6, the medium was changed to BDM supplemented with 2% conditioned medium derived from the supernatants of AFT024-mIL3, AFT024-mIL6, AFT024-hFlt3L, and AFT024-mSCF cell cultures. Doxycycline (1 μg/mL, Sigma) was added on Day 6. The medium was replaced every other day. The plates were seeded with OP9-DL1 cells (20,000 cells/well, 12-well plate) 12 h prior to the hematopoietic maturation step in EM (α-MEM, 15% FBS (HyClone), 200 μg/mL iron-saturated transferrin, 0.1 mM β-mercaptoethanol, 1% GlutaMAX, 50 μg/mL ascorbic acid, 2% conditioned medium derived from supernatants of AFT024-mIL3, AFT024-hFlt3L, and AFT024-mSCF cell cultures and 1 μg/mL doxycycline). Then, 1000–3000 sorted iHECs were seeded into each well for hematopoietic maturation. Half of the EM was replaced every two days.

### Transplantation of iHPCs

Eight- to ten-week-old μMT mice, C57BL/6 mice, and *Rag1*^−/−^ mice were sublethally irradiated (5 Gy, 6.5 Gy, and 3.5 Gy, respectively), by an X-ray irradiator (RS2000, Rad Source Inc.). A total of 5 million *iRunx1-Hoxa9-Lhx2*-ESC-derived iHPCs were injected into each irradiated μMT mouse, C57BL/6 mouse, or *Rag1*^−/−^ mouse via retro-orbital veins. In addition, 3 million *iRunx1-Lhx2*-ESC-derived iHPCs were injected into each irradiated *Rag1*^−/−^ mouse via retro-orbital veins. The mice were fed water containing doxycycline (1 mg/mL) to induce the generation of B lymphocytes.

### Flow cytometry and cell sorting

Single-cell suspensions were prepared in phosphate-buffered saline (PBS) supplemented with 2% fetal bovine serum (FBS) and filtered through a 70 μm filter. Single cells were blocked with an anti-Fc (CD16/32) (BioLegend) antibody and then stained with related antibodies. The following antibodies were used: c-Kit (2B8, eBioscience), CD31 (390, eBioscience), CD41 (eBioMWReg30, eBioscience), CD45 (30-F11, eBioscience), CD201 (eBio1560, eBioscience), CD2 (RM2-5, eBioscience), CD3 (145-2C11, eBioscience), CD4 (GK1.5, eBioscience), CD8a (536.7, eBioscience), B220 (RA3-6B2, eBioscience), B220 (RA3-6B2, BioLegend), Mac1 (M1/70, eBioscience), Mac1 (M1/70, BioLegend), NK1.1 (PK136, BioLegend), NK1.1 (PK136, eBioscience), Ter119 (TER-119, eBioscience), Gr1 (RB6-8C5, eBioscience), IgM (II/41, eBioscience), IgD (11-26 c.2a, BioLegend), Sca-1 (D7, eBioscience), CD19 (eBio1D3, eBioscience), CD23 (B3B4, BioLegend), CD21/35 (7G6,BD Biosciences), CD43 (eBioR2/60, eBioscience), CD24 (M1/69, BioLegend), Ly-51 (6C3, BioLegend), CD93 (AA4.1, BioLegend), CD5 (53-7.3, BioLegend), CD138 (281-2, BioLegend), CD38 (90, BioLegend), GL7 (GL-7, eBioscience), IgG1(RMG1-1, BioLegend), CD22 (Cy34.1, BD Biosciences), MHC II (M5/114.15.2, BioLegend), streptavidin Alexa Fluor^®^ 700 (Invitrogen), streptavidin PE-Cy7 (BioLegend), and NP-PE (Biosearch Technologies). The cells were resuspended in DAPI solution (Sigma) or PI solution (BioLegend) and analyzed with a Fortessa cytometer (BD Biosciences). The cells were sorted using an Arial III cytometer (BD Biosciences). The flow cytometry data were analyzed with FlowJo.

### Immunization and serum collection

T cell-dependent antigen immunization was performed as described previously [24, 25]. Briefly, iB-μMT mice 4 weeks after transplantation and μMT mice were immunized i.p. with 100 μg 4-hydroxy-3-nitrophenyl acetyl (NP)-CGG (Biosearch Technologies) in alum (Thermo Fisher Scientific) at a volume ratio of 1:1 (200 μl/mouse). To induce recall responses, mice were challenged with 50 μg NP-CGG at week 16 after primary immunization (100 μl/mouse). Sera were collected from each group on Day 0, Day 7, Day 14, Day 21, Day 111, Day 116, Day 121, and Day 126 after primary immunization. Antigen-specific antibodies were measured by ELISA.

### ELISA

For basal serum Ab (IgM/IgG1/IgG2b/IgG2c/IgG3/IgA) measurement, microtiter plates were coated with goat anti-mouse Ig (5 μg/ml, Southern Biotech) overnight at 4 °C. For NP-specific Ab measurement, NP(27)-BSA (Biosearch Technology) or NP(9)-BSA (high affinity) (Biosearch Technology) was used as the capture antigen. Then, nonspecific binding was blocked with 0.5% BSA in PBS for 2 h at 37 °C. Diluted serum samples were incubated in plates for 1 h at 37 °C. Plates were incubated for 1 h with goat anti-mouse IgA-HRP, goat anti-mouse IgM-HRP, goat anti-mouse IgG1-HRP, goat anti-mouse IgG2b-HRP, goat anti-mouse IgG2c-HRP, and goat anti-mouse IgG3-HRP (all from Southern Biotech) and then for 15–30 mins with 100 µl/well TMB (BioLegend) substrate solution, followed by incubation with 50 µL 2 N H2SO4 to stop the reaction. Absorbance values were read at 450 nm using a microplate reader (Cytation5, BioTek).

### BCR sequencing

For BCR sequencing, 100,000 naïve FO B cells were sorted from the spleen of one iB-μMT mouse 4 weeks after transplantation and one C57BL/6 (B6) mouse. The sorted naïve FO B cells were gated by CD45^+^CD19^+^IgD^++^IgM^+^CD23^++^CD21^+^CD3^-^CD4^−^CD8^−^Ter119^−^Gr1^−^Mac1^−^NK1.1^−^CD138^−^. Total RNA was extracted from naïve FO B cells using TRIzol (MRC). 5’ RACE was performed with a SMARTer RACE cDNA Amplification Kit (Clontech). IgG/IgK/IgL NGS libraries were made by using the NEBNext Ultra DNA Library Prep Kit for Illumina (NEB). Libraries were sequenced on the Illumina MiSeq 2 × 300 platform. The raw data (fastq files) were generated using Illumina bcl2fastq software and uploaded to the Gene Expression Omnibus public database. The B cell receptor repertoires were aligned and assembled using MiXCR software (version 3.0.13). The BCR IgH/IgL/IgK clonotypes were exported with the parameter ‘--chains’ in the exportClones command of MiXCR [26]. The exported clonotypes were visualized in the form of a chord diagram using VDJtools software (version 1.2.1) [27].

### scRNA-seq and data analysis

Fifty thousand sorted early bone marrow regenerative progenitors (GFP^+^CD45^+^CD3^−^CD4^−^CD8^−^Ter119^−^Gr1^−^Mac1^−^NK1.1^−^) taken from iB-μMT mice (*n* = 4) on Day 7.5 after transplantation were used for scRNA-seq. Droplet-based scRNA-seq datasets were produced using a Chromium system (10x Genomics, PN120263) following the manufacturer’s instructions. Droplet-based scRNA-seq datasets were aligned and quantified using the CellRanger software package (version 4.0.0) and subjected to Seurat (version 3.2.3) [28] for further analysis. To pass quality control, cells were required to have less than 60,000 raw reads mapped to nuclear genes, at least 2000 genes detected, and less than 10% of the mapped reads mapped to mitochondrial genes. Ultimately, 7977 cells passed the quality control. To rule out the effects of cell cycle variances, we performed simple linear regression against the cell cycle score calculated by CellCycleScoring. Then, PCA was performed by RunPCA using 2000 highly variable genes, and the top 20 PCs were used for UMAP analysis. Clusters were detected using FindClusters with parameter settings dims = 1:20 and resolution = 0.08. Violin and dot plots for gene expression were plotted using the VlnPlot function of Seurat and the ggplot2 package. Upregulated genes were identified for each cluster using the Wilcoxon rank sum test with the parameters min.pct = 0.5 and logfc.threshold = 0.25 implemented in Seurat. Heatmaps for average gene expression were plotted by pheatmap (version 1.0.12). Gene ontology enrichment analysis (for biological processes) was performed with the upregulated genes of each cluster by clusterProfiler with a BH-adjusted *p* value cutoff = 0.05 (version 3.14.3) [29].

Droplet-based single-cell RNA-seq of CD19^+^ B lymphoid progenitor cells was downloaded from the Gene Expression Omnibus repository (GSE114793). In addition, projection of cells from the induced B lymphoid progenitor cells in our study onto wild-type mouse CD19^+^ B lymphoid progenitor cells (pro-B, large pre-B, and small pre-B populations) was performed using the Seurat package. Before integrating data, the effect of cell cycle gene expression was removed. Two datasets were integrated using Seurat’s integration function. First, anchors were identified with the FindIntegrationAnchors function, and then the IntegrateData function was used with dim = 1:30. The standard workflow for UMAP dimensionality reduction was performed using the top 10 PCs. Furthermore, each cell was assigned an identity by the FindTransferAnchors and TransferData functions using wild-type pro-B, large pre-B, and small pre-B populations.

### Statistics

Data analyses were performed using GraphPad Prism. All data are expressed as the mean, and the specific number (n) for each dataset is detailed in the figure legends. All statistical analyses were performed by independent-sample Student’s *t* test and Mann–Whitney *U* tests (SPSS software). The results are notated as follows: NS, not significant; *P < 0.05; **P < 0.01; ***P < 0.001.

## Results

### Transplantation of iHPCs derived from a *Runx1-p2a-Hoxa9-t2a-Lhx2*-ESC line preferentially gives rise to B lymphopoiesis in B cell-deficient mice

**Fig. 1:**
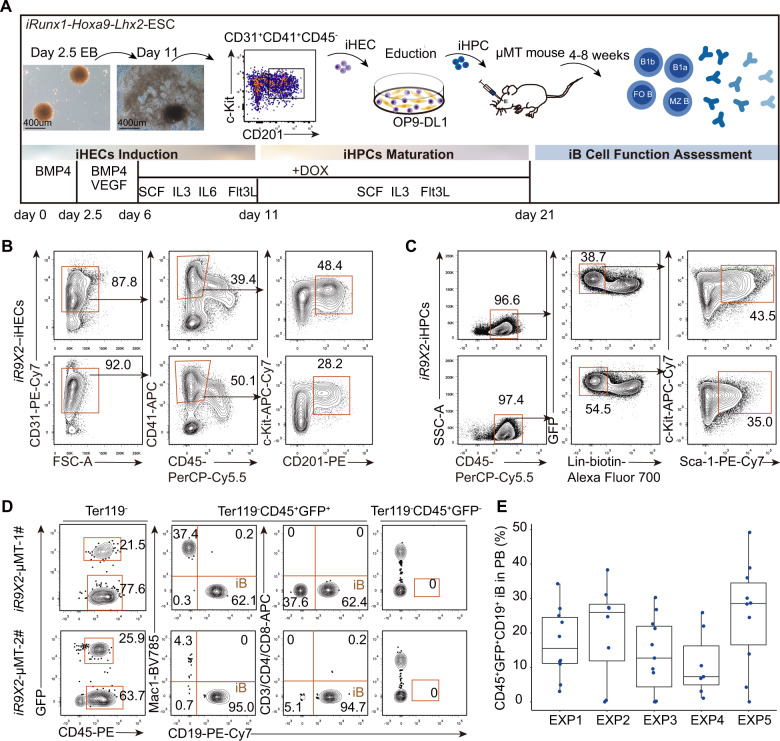
Reconstitution of B cells in vivo from iRunx1-p2a-Hoxa9-t2a-Lhx2-modified embryonic stem cells. **A** Schematic diagram of B cell regeneration from *iRunx1-p2a-Hoxa9-t2a-Lhx2* ESCs. ESC differentiation was initiated by embryoid body formation (EB). On Day 2.5, EBs were replanted into gelatinized plates and differentiated into iHECs with cytokines. On Day 11, iHECs were replated on OP9-DL1 stroma with cytokines for differentiation into hematopoietic progenitor cells. Inducible expression of *Runx1*, *Hoxa9*, and *Lhx2* was achieved with addition of doxycycline (1 μg/mL, Sigma) from Day 6 to Day 21. On Day 21, 5 million *iR9X2*-ESC-derived iHPCs were injected into each sublethally irradiated (5 Gy) μMT mouse via retro-orbital veins. The mice were fed water containing doxycycline (1 mg/mL) to induce the generation of B lymphocytes. After transplantation, B cell production was analyzed by flow cytometry, and B cell function was evaluated. **B** Strategies for sorting the iHEC population taken from *iR9X2*-ESCs on Day 11. EB-derived iHECs (CD31^+^CD41^+^CD45^−^c-Kit^+^CD201^+^) were sorted by flow cytometry. Two representative plots from five independent experiments are shown. **C** Immunophenotypes of induced hematopoietic progenitor cells taken from iHECs after ten days of education. iHECs were cocultured with OP9-DL1 cells for 10 days to generate iHPCs, and fluorescence-activated cell sorting (FACS) analysis of the iHPCs showed a Lin^−^c-Kit^+^Sca-1^+^ phenotype. Two representative plots from five independent experiments are shown. Lin^−^ was defined as CD2^−^CD3^−^CD4^−^CD8^−^Mac1^−^Gr1^−^Ter119^−^B220^−^NK1.1^−^. **D** *iR9X2*-ESC-derived B (iB) cells in the peripheral blood (PB) of μMT mice were analyzed by flow cytometry 4 weeks after transplantation. Five million iHEC-derived hematopoietic progenitors were transplanted into each sublethally irradiated μMT mouse (5 Gy). The mice were fed water containing doxycycline (1 mg/mL) to induce the generation of B lymphocytes. iHPC-derived hematopoietic cells (GFP^+^CD45^+^Mac1^+^ myeloid cells, GFP^+^CD45^+^CD3/CD4/CD8^+^ T cells, and GFP^+^CD45^+^CD19^+^ B cells) were analyzed 4 weeks after transplantation. GFP^−^CD45^+^CD19^+^ cells of the same *iR9X2*-μMT recipients are shown as controls. Two representative mice from five independent experiments are shown. **E** Summarized findings of iB cells in the PB of individual μMT mice from five independent experiments. Forty-six μMT mouse recipients were analyzed at week 4 after transplantation of ESC-derived iHPCs. The box plot shows the percentage of CD45^+^GFP^+^CD19^+^ iB cells in the PB. The percentages were visualized by ggplot2 (R package). One point represents one mouse

To induce B cell lymphopoiesis, we followed a two-step method of testing transcription factor combinations [22, 23]. An inducible expression cassette of *Runx1-p2a-Hoxa9-t2a-Lhx2* was introduced into the *Rosa26* locus of a GFP-transgenic mouse embryonic stem cell line (C57BL/6 background) by homologous recombination to establish the *iR9X2*-ESC cell line (Fig. S1A). Conditional expression of exogenous *Runx1*, *Hoxa9*, and *Lhx2* was confirmed in the presence of doxycycline (Fig. S1B). Following the protocol for hematopoietic progenitor cell induction from ESCs in vitro [22, 23] (Fig. 1A), BMP4 and VEGF were used to induce mesoderm differentiation and hemangioblast formation from the embryoid body. AFT024-(mSCF/mIL3/mIL6/hFlt3L) cell line culture supernatants were used as conditioned medium (CM) for the in vitro induction of iHECs and subsequently iHPCs, as AFT024 CM is beneficial for the generation of iHPCs in vitro [30]. iHECs (CD31^+^CD41^+^CD45^−^c-Kit^+^CD201^+^) phenotypically resembling embryonic pre-HSCs [31] were generated from *iR9X2*-ESCs on Day 6 to Day 11 in the presence of doxycycline (Fig. 1B). The iHECs cocultured with OP9-DL1 feeder cells were further educated into Lin^−^c-Kit^+^Sca-1^+^ iHPCs from Day 11 to Day 21 in the presence of doxycycline (Fig. 1C). To assess the engraftment potential of these iHPCs, we transplanted 5 million *iR9X2*-ESC-derived iHPCs (*iR9X2*-iHPCs) on Day 21 into sublethally irradiated (5 Gy) B cell-deficient μMT mice (*iR9X2*-μMT mice) that received continuous doxycycline water feeding after transplantation. Four weeks after transplantation, we observed donor-derived GFP^+^CD45^+^CD19^+^ B cells but no GFP^+^CD45^+^CD3/CD4/CD8^+^ T cells and no GFP^−^CD45^+^CD19^+^ B cells in the peripheral blood (PB) of *iR9X2*-μMT mice transplanted with iHPCs (Fig. 1D). We also observed donor-derived GFP^+^CD45^+^Mac1^+^ myeloid cells in the PB of recipients four weeks after transplantation (Fig. 1D; Fig. S1C). However, the donor-derived myeloid cells were transient and barely detectable in the PB of *iR9X2*-μMT mice at week 8 after transplantation (Fig. S1C). Several independent experiments indicated that the engraftment rate of *iR9X2*-ESC-derived iHPCs was 91.3% (42/46 mice), resulting in a total average of 17.7% donor B cells in the PB of μMT recipients (*n* = 46) at week 4 after transplantation (Fig. 1E). Importantly, we observed *iRunx1-p2a-Hoxa9-t2a-Lhx2-ESC*- derived CD45^+^CD19^+^ B cells in the PB of C57BL/6 and *Rag1*^−/−^ recipients after transplantation (Fig. S1D, E). Thus, inducible expression of *Lhx2*, *Hoxa9*, and *Runx1* leads to ESC differentiation toward hematopoietic progenitors preferentially producing B lymphopoiesis.

**Fig. 2:**
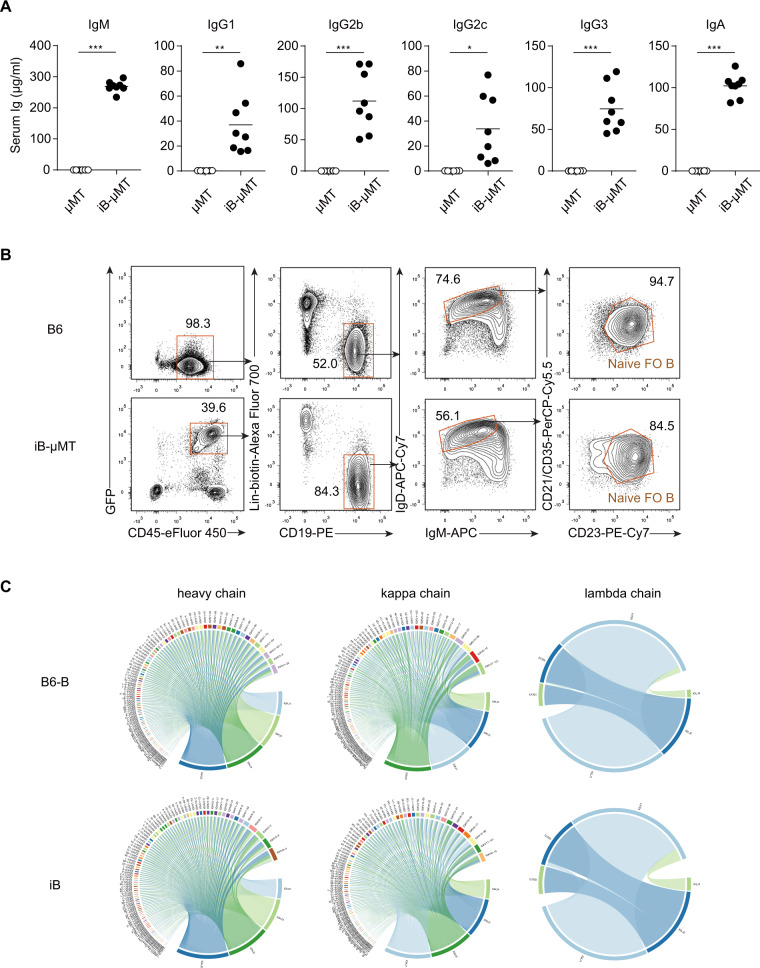
Basal levels of serum Ig in iB-μMT mice and the BCR repertoires of regenerative naïve FO B cells. **A** Serum Ig levels in iB-μMT mice and μMT mice (*n* = 8 per group) were measured by ELISA. Sera were collected from iB-μMT mice (4 to 6 weeks after transplantation) and μMT mice. The different isotypes of antibodies (IgM/IgG1/IgG2b/IgG2c/IgG3/IgA) were measured by ELISA. Each symbol represents an individual mouse; the small horizontal lines indicate the means. *P < 0.05, **P < 0.01, and ***P < 0.001 (independent-sample Student’s *t* test). **B** Naïve follicular B (FO B) cell sorting strategy for BCR sequencing. One iB-μMT mouse was sacrificed 4 weeks after transplantation, and one C57BL/6 (B6) mouse was sacrificed as a control. From the spleen, naïve FO B cells were sorted based on CD19^+^IgD^++^IgM^+^CD23^++^CD21^+^Lin^−^(CD3^−^CD4^−^CD8^−^Ter119^−^Gr1^−^Mac1^−^NK1^.^1^−^CD138^−^) surface expression. **C** Chord diagram of IgH/IgK/IgL diversity in iB cells. Aliquots of 100,000 sorted naïve FO B cells from the spleen of an iB-μMT mouse and a B6 mouse were used as cell inputs for BCR sequencing. The BCR IgH/IgL/IgK clonotypes were visualized in the form of a chord diagram using VDJtools software

To determine whether iB cells possess antibody production ability, we quantified preimmune Ig isotype levels in sera from *iR9X2*-μMT (iB-μMT) mice and μMT mice. We found significant levels of serum IgM, IgG1, IgG2b, IgG2c, IgG3, and IgA in iB-μMT mice 4 to 6 weeks after iHPC transplantation (Fig. 2A), whereas serum Ig isotypes could not be detected in μMT mice. Eighteen to 40 weeks after iHPC transplantation, we could still detect significant preimmune Ig isotype levels in the sera from iB-μMT mice (Fig. S2). The diversity of BCRs generated by the rearrangement of V(D)J gene segments in B cells [32] is essential for humoral immune protection, as highly diverse antibody repertoires are capable of recognizing a plethora of foreign antigens. To further assess the BCR repertoires of iB cells, we sorted naïve FO B cells (CD19^+^IgD^++^IgM^+^CD23^++^CD21^+^Lin^−^) taken from the spleen of one iB-μMT mouse at week 4 after transplantation and one C57BL/6 (B6) mouse for BCR deep sequencing (Fig. 2B). Aliquots of 100,000 sorted naïve FO B cells were used as cell inputs for BCR sequencing. BCR clonotype profiling using MiXCR [26] captured abundant BCR sequences among the sorted naïve FO B cells isolated from the spleen of the iB-μMT mouse,  which resembled their natural cell counterparts (Fig. 2C). Collectively, these data indicate that the humoral immune system is successfully reconstituted from the *iR9X2*-ESC source with functional iB cells that express highly diverse BCR repertoires.

### The regenerative B cell hierarchy shows a similar trajectory of natural B cell lymphopoiesis

**Fig. 3:**
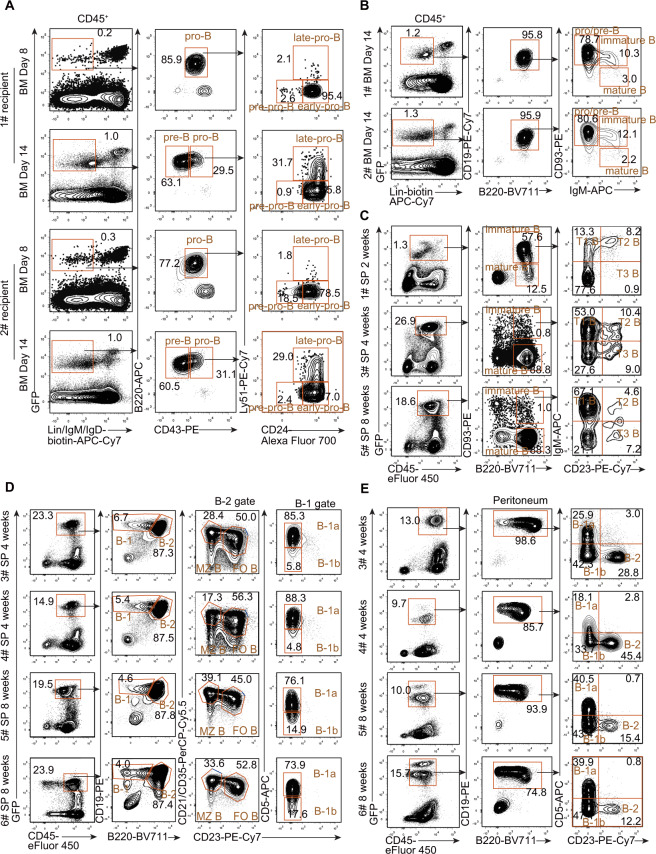
Cellular hierarchy of iB cell development in iB-μMT mice. **A** Flow cytometry analysis of pro-B cells and pre-B cells in the bone marrow of iB-μMT mice. Each μMT recipient was transplanted with five million *iR9X2*-iHPCs collected on Day 21. The tibias of representative mice were amputated and analyzed 8 days after transplantation; the same mice were further sacrificed and analyzed 14 days after transplantation. Pro-B (GFP^+^Lin^−^IgM^−^IgD^−^B220^+^CD43^+^) and pre-B (GFP^+^Lin^−^IgM^−^IgD^−^B220^+^CD43^−^) cells from two representative mice are shown. Pro-B cells were further divided into three subsets, pre-pro-B, early pro-B, and late pro-B cells, on the basis of the expression of CD24 and Ly-51. Lin^−^ was defined as Ter119^−^Mac1^−^Gr1^−^NK1.1^−^CD3^−^CD4^−^CD8^−^. **B** Flow cytometry analysis of pro/pre-B cells, immature B cells, and mature B cells in the bone marrow of iB-μMT mice 14 days after transplantation. Each μMT mouse recipient was transplanted with five million *iR9X2*-iHPCs collected on Day 21. Data from two representative mice are shown. Lin^−^ was defined as Ter119^−^Mac1^−^Gr1^−^NK1.1^−^CD3^−^CD4^−^CD8^−^. **C** Transitional B cells were analyzed by flow cytometry from the spleens of iB-μMT mice. Each recipient was transplanted with five million *iR9X2*-iHPCs collected on Day 21. iB-μMT mice were sacrificed and analyzed 2 weeks, 4 weeks, and 8 weeks after transplantation. Representative FACS plots from three iB-μMT mice are shown. **D** Phenotypic analysis of B-1a, B-1b, follicular B (FO B), and marginal zone B (MZ B) cells in the spleens of iB-μMT mice 4 weeks and 8 weeks after transplantation. Each recipient was transplanted with five million *iR9X2*-iHPCs collected on Day 21. Data from four representative mice are shown. **E** Phenotypic analysis of B-1a, B-1b, and B-2 cells in the peritoneal cavity of iB-μMT mice 4 weeks and 8 weeks after transplantation. Each recipient was transplanted with five million *iR9X2*-iHPCs collected on Day 21. Data from four representative mice are shown

We further observed donor-derived GFP^+^CD45^+^CD19^+^ iB cells in the bone marrow and spleen of μMT recipients (Fig. S3A, B). To analyze the immunophenotypes of the regenerative B lymphocytes in iB-μMT mice, we first detected induced pro-B cells and pre-B cells in the bone marrow, where B lymphopoiesis originates. GFP^+^Lin^−^B220^+^CD43^+^ pro-B cells were detected in the bone marrow of iB-μMT mice on Day 8 after *iR9X2*-iHPC transplantation (Fig. 3A). Induced pro-B cells could be separated into pre-pro-B (fraction A), early pro-B (fraction B), and late pro-B/early pre-B (fraction C/C′) cells according to Hardy’s criteria [33] (Fig. 3A). The induced pre-B cells, which lacked CD43 expression and arose from pro-B cells, appeared in the bone marrow of the same iB-μMT mice on Day 14 after *iR9X2*-iHPC transplantation (Fig. 3A). The majority of the induced pro-B cells were in the early pro-B fraction on Day 8 and further progressed into the late pro-B/early pre-B fraction on Day 14. CD93^+^IgM^+^ immature B cells and CD93^−^IgM^+^ mature B cells arose in the central bone marrow of iB-μMT mice on Day 14 after *iR9X2*-iHPC transplantation (Fig. 3B). GFP^+^CD45^+^CD93^+^B220^+^ transitional B cells, which were early emigrant cells from the bone marrow, were detected in the spleen of iB-μMT mice and could be divided into the T1 population, T2 population, and T3 population according to the expression of surface IgM and CD23. Immature B cells further developed into GFP^+^CD45^+^CD93^−^B220^+^ mature B cells in the spleen of iB-μMT mice (Fig. 3C; Fig. S3C). Interestingly, the majority of the iB cells in the spleen were transitional B cells at week 2 after transplantation and then further progressed into mature B cells at week 4 and week 8 (Fig. 3C; Fig. S3C). Importantly, all mature B cell subsets (B-1a, B-1b, FO B, and MZ B cell subsets) existed in the spleen of iB-μMT mice at week 4 and week 8 after transplantation (Fig. 3D). The presence of CD19^+^B220^−/low^CD23^-^ B-1 cells was further confirmed by analysis of the positive surface marker CD43 in the spleen (Fig. S3D). Induced B-1 and B-2 cells were also detected in the peritoneal cavity of iB-μMT mice at week 4 and week 8 after transplantation (Fig. 3E). Furthermore, we could still observe all mature iB cell subsets (B-1a, B-1b, FO B, and MZ B cell subsets) in the spleen and peritoneum of iB-μMT mice at week 40 after transplantation (Fig. S3E, F), although pro/pre-B and immature B cells were barely detected in the bone marrow of iB-μMT mice at week 6 after transplantation (Fig. S3G), indicating a long lifespan of mature iB cells. Taken together, these data indicate that *iR9X2*-ESC-derived iHPCs reconstitute B lymphopoiesis in vivo in a spatiotemporal kinetic distribution pattern resembling natural B cell development.

### Single-cell RNA-seq reveals the transcriptome features of regenerative pro-B and pre-B cells

**Fig. 4:**
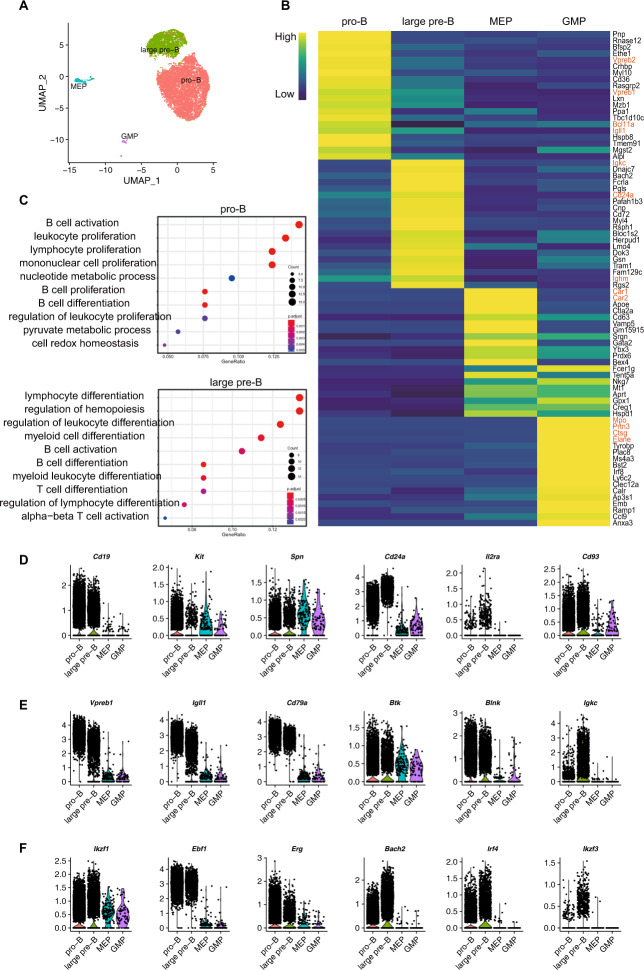
Single-cell transcriptomic characterization of regenerative pro-B and pre-B cells. **A** UMAP visualization of early bone marrow regenerative progenitor cells in iB-μMT mice. Fifty thousand bone marrow cells were sorted based on GFP^+^CD45^+^CD3^−^CD4^−^CD8^−^Ter119^−^Gr1^−^Mac1^−^NK1.1^−^ surface expression for sequencing on Day 7.5 after transplantation. Of these, 7977 cells were retained for UMAP analysis. To rule out the effects of cell cycle variances, we performed simple linear regression against the cell cycle score calculated by CellCycleScoring. Then, PCA was performed by RunPCA using 2000 highly variable genes, and the top 20 PCs were used for UMAP analysis. Clusters were detected using FindClusters with parameter settings dims = 1:20 and resolution = 0.08. One point represent one cell. **B** Heatmap showing the average expression of the top 20 differentially expressed genes in each cluster of early bone marrow regenerative progenitor cells from iB-μMT mice. The average expression value is the scaled average expression. Upregulated genes were identified for each cluster using the Wilcoxon rank sum test with the parameters min.pct = 0.5 and logfc.threshold = 0.25 implemented in Seurat. One cluster is shown per column. The expression values were *z* score transformed by Seurat packages. **C** Gene ontology (GO) enrichment analysis of the differentially expressed genes in pro-B and large pre-B clusters. Gene ontology enrichment analysis (for biological processes) was performed with the upregulated genes of each cluster by clusterProfiler with a BH-adjusted *p* value cutoff = 0.05. Each symbol represents a GO term (noted in the plot); the color indicates the adjusted *P* value (Padj (significance of the GO term); bottom key), and the symbol size is proportional to the number of genes (top key). **D** Violin plots showing the expression profile of pro-B cell- and large pre-B cell-related surface marker genes (*CD19*, *Kit*, *Spn*, *Cd24a*, *Il2ra*, and *Cd93*). The expression value of each gene was normalized by the “LogNormalize” method and visualized by the VlnPlot function of Seurat. **E** Violin plots showing the expression profile of selected pre-BCR- and BCR formation-related marker genes (*Vpreb1*, *Igll1*, *CD79a*, *Btk*, *Blnk*, and *Igkc*). The expression value of each gene was normalized by the “LogNormalize” method and visualized by the VlnPlot function of Seurat. **F** Violin plots showing the expression profile of selected early B cell development-related transcription factors (*Ikzf1*, *Ebf1*, *Erg*, *Bach2*, *Irf4*, and *Ikzf3*). The expression value of each gene was normalized by the “LogNormalize” method and visualized by the VlnPlot function of Seurat

To characterize the transcriptome landscape of the early bone marrow regenerative progenitors in iB-μMT mice, we performed single-cell RNA-Seq using sorted GFP^+^CD45^+^CD3^−^CD4^−^CD8^−^Ter119^−^Gr1^−^Mac1^−^NK1.1^−^ cells taken from the bone marrow of iB-μMT mice on Day 7.5 after *iR9X2*-iHPC transplantation (Fig. S4A). Then, the scRNA-seq datasets were processed, and 4 clusters of a total of 7977 single cells, including pro-B cells, large pre-B cells, megakaryocyte/erythrocyte progenitors (MEPs), and granulocyte/macrophage progenitors (GMPs), were identified and visualized using UMAP based on their unique gene expression signatures (Fig. 4A). Two clusters were identified as B cell progenitors based on their upregulated expression of genes encoding proteins involved in B cell activation and B cell differentiation (*Vpreb2, Vpreb1, Bcl11a, Igll1, Igkc, Cd24a, Ighm*) (Fig. 4B, C), the surface marker-encoding gene *Cd19* (Fig. 4D), which first appears around the time of immunoglobulin gene rearrangement [34], and *Cd93* (Fig. 4D), which marks early B lineage cells [35, 36]. Pro-B cells (5754 single cells) were identified by their expression of surface marker-encoding genes, including *Kit*, *Spn*, and *Cd24a* (Fig. 4D), while large pre-B cells (2013 single cells) were characterized by loss of *Kit* expression (Fig. 4D; Fig. S4C), expression of *Il2ra* and *Igkc* (Fig. 4D, E; Fig. S4C, D), and upregulated expression of the surface marker gene *Cd24a* [33, 37] (Fig. 4D; Fig. S4D). During early B cell development, recombinase-activating genes (*Rag1/Rag2*) and DNA nucleotidylexotransferase (*Dntt*), which are essential for VDJ recombination [38] at the pro/pre-B cell stage, were expressed (Fig. S4B). After immunoglobulin heavy chain rearrangement, the expression of *Dntt* was silenced at the large pre-B cell stage [39, 40] (Fig. S4B–D). And the pre-BCR complex, which was composed of immunoglobulin μ heavy chain, surrogate light chain encoded by VpreB and λ5 (Fig. 4E; Fig. S4B), and the signaling molecules Igα/CD79a and Igβ/CD79b (Fig. 4E; Fig. S4B), started assembling. The expression of pre-BCR  components is crucial for pre-B cell differentiation as loss of any component will arrest the pro-B cell transition to the pre-B cell stage [41–44]. Accordingly, the pre-BCR signaling resulted in downregulation of *Rag1* [45] (Fig. S4B, D) and proliferation of large pre-B cells [46, 47]. The silence of VpreB and λ5 (Fig. 4E; Fig. S4B, D) further terminated large-pre B cell expansion and drived differentiation into the small pre-B cell stage [48]. In addition, pro-B and large pre-B cells expressed Bruton’s tyrosine kinase (*Btk*) and B cell linker protein (*Blnk*) (Fig. 4E), which are key cytoplasmic component genes of the pre-BCR signaling pathway [49, 50]. Transcription factor genes involved in the regulatory network of early B cell development, such as *Ikzf1*, *Spi1*, *Tcf3*, *Foxo1*, *Ebf1*, *Bcl11a*, and *Pax5*, were widely expressed among pro-B and large pre-B populations (Fig. 4F; Fig. S4B), and loss of any one results in an arrest of B cell differentiation [51–57]. In addition, pro-B cells showed abundant expression of the transcription factor *Erg*, which was reduced at the large pre-B stage (Fig. 4F; Fig. S4C), suggesting that it is an exquisitely stage-specific regulator of early B cell development [58]. The transcription factor *Bach2* was widely expressed and upregulated in large pre-B cells (Fig. 4F; Fig. S4C, D), which is required for mediating negative selection at the pre-BCR checkpoint [59]. The expression of the transcription factor *Irf4* was upregulated (Fig. 4F; Fig. S4C, D), and the expression of the transcription factor *Ikzf3* was induced (Fig. 4F; Fig. S4C) upon pre-BCR signaling [60, 61] at the large pre-B cell stage, and which play a critical role in further downregulating pre-BCR and suppressing large pre-B cell expansion in the transition from large pre-B to small pre-B cells [62, 63]. In addition, two small clusters of MEP cells and GMP cells (141 single cells and 69 single cells, respectively), were marked by high expression of carboxylate reductases (*Car1* and *Car2*) [64] and a number of granule genes, including myeloperoxidase (*Mpo*), neutrophil elastase (*Elane*), proteinase 3 (*Prtn3*), and cathepsin G (*Ctsg*) (Fig. 4B), which explains the transient wave of myeloid lineage cells in iB-μMT mice. To compare iB cells with their wild-type B lymphocyte counterparts, the IntegratedData and TransferData functions of Seurat were implemented to project iB cell data onto wild-type pro-B, small pre-B, and large-preB cell data derived from scRNA-seq data (GSE114793) of wild-type B lymphocytes (live LIN^−^CD19^+^IgM^−^IgD^−^, LIN-antibodies = Gr1, Ter119, CD3, Mac1, CD11c, NK1.1). The UMAP dimensionality reduction of the integrated data is presented in Fig. S5A. In the projection result, 92.08% of induced pro-B cells (5298/5754) were projected as control pro-B cells, and 90.01% of induced large pre-B cells (1812/2013) were projected as control large pre-B cells (Fig. S5B). Thus, the projection results showed that induced pro-B and induced large pre-B cells resemble natural pro-B and large pre-B cells. Overall, the large-scale single-cell transcriptome features demonstrate that *iR9X2*-iHPCs robustly differentiate into early B cell progenitors at as early as Day 7.5 after transplantation.

### Regenerative B cells produce an adaptive immune response and form long-term immune memory

**Fig. 5:**
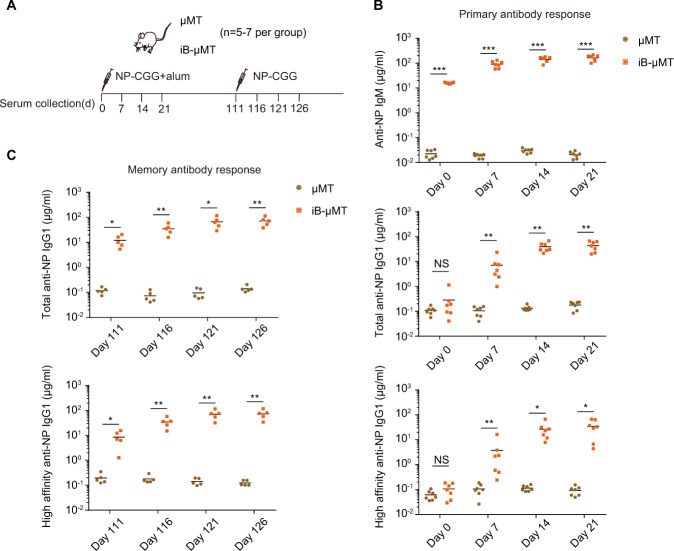
Antibody response to T cell-dependent antigen (NP-CGG) in iB-μMT mice. **A** Schematic diagram of iB-μMT mice and μMT mice immunized with NP-CGG and from which sera were collected at the indicated time points. iB-μMT mice (*n* = 7) and μMT mice (*n* = 7) were given primary intraperitoneal immunization with 100 μg NP-CGG in alum on Day 0. Then, iB-μMT mice (*n* = 5) and μMT mice (*n* = 5) were given a secondary challenge with 50 μg NP-CGG on Day 111 after primary immunization. Sera were collected on Day 0, Day 7, Day 14, Day 21, Day 111, Day 116, Day 121, and Day 126 after primary immunization. **B** T cell-dependent primary antibody response in iB-μMT mice. iB-μMT mice (*n* = 7) and μMT mice (*n* = 7) were immunized with 100 μg NP-CGG in alum on Day 0. Anti-NP IgM (top), total anti-NP IgG1 (middle), and high-affinity anti-NP IgG1 (bottom) antibodies in the sera collected at the indicated time points were measured by ELISA. Each symbol represents an individual mouse, and the horizontal lines indicate the mean values. NS, not significant, *P < 0.05, **P < 0.01, and ***P < 0.001 (independent-sample Student’s *t* test and Mann–Whitney *U* test). **C** Production of memory antibodies in iB-μMT mice. Sera were collected from iB-μMT mice (*n* = 5) and μMT mice (*n* = 5) at the indicated time points after boost with 50 μg NP-CGG. Levels of total anti-NP IgG1 and high-affinity anti-NP IgG1 were measured by ELISA. Each symbol represents a mouse, and the horizontal lines indicate the mean values. *P < 0.05, **P < 0.01, and ***P < 0.001 (independent-sample Student’s *t* test and Mann–Whitney *U* test)

**Fig. 6:**
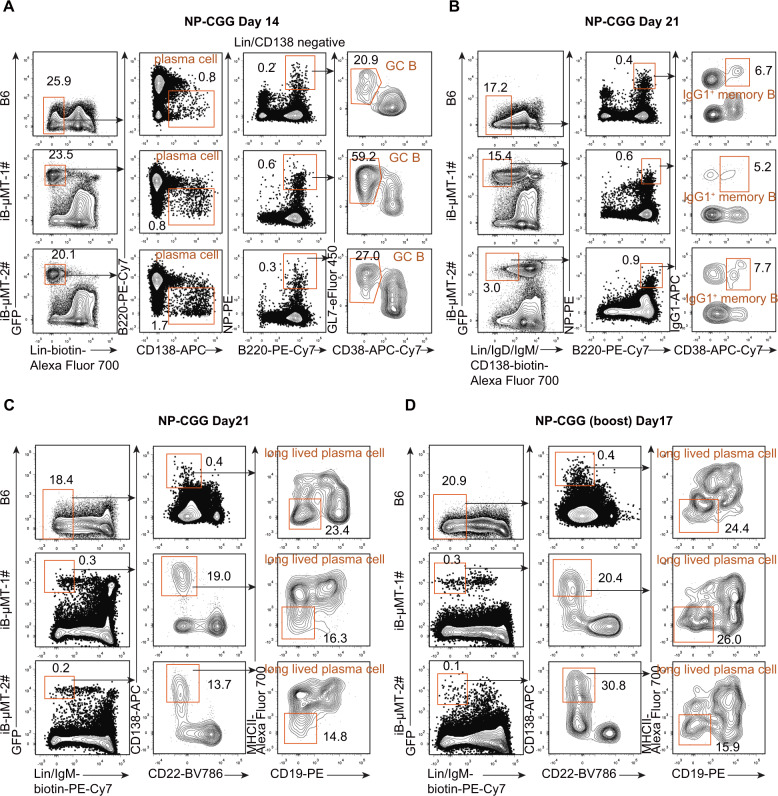
Normal germinal center (GC) B, memory B, and plasma cell formation in iB-μMT mice. **A** Plasma cells and antigen-specific GC B cells in the spleens of iB-μMT mice. Spleen cells were isolated from B6 and iB-μMT mice 14 days after primary intraperitoneal immunization with 100 μg NP-CGG in alum. Plasma cells (Lin^−^B220^low/−^CD138^+^) and antigen-specific GC B cells (Lin^−^B220^+^CD138^−^NP^+^GL7^+^CD38^−^) were analyzed by flow cytometry. Data from one representative B6 mouse and two representative iB-μMT mice are shown. Lin^−^ was defined as Ter119^−^Mac1^−^Gr1^−^NK1.1^−^CD3^−^CD4^−^CD8^−^. **B** Antigen-specific class-switched IgG1^+^ memory B cells in the spleens of iB-μMT mice. Spleen cells were isolated from B6 and iB-μMT mice 21 days after primary intraperitoneal immunization with 100 μg NP-CGG in alum. Representative flow cytometry plots of antigen-specific class-switched IgG1^+^ memory B cells (Lin^−^IgD^−^IgM^−^CD138^−^B220^+^NP^+^CD38^+^IgG1^+^) from one B6 mouse and two iB-μMT mice are shown. Lin^−^ was defined as Ter119^−^Mac1^−^Gr1^−^NK1.1^−^CD3^−^CD4^−^CD8^−^. **C**, **D** Long-lived plasma cells in the bone marrow of iB-μMT mice. Long-lived plasma cells (Lin^−^IgM^−^CD138^+^CD22^−^CD19^−^MHCII^−^) in the bone marrow of B6 and iB-μMT mice were analyzed by flow cytometry. Representative plots from B6 and iB-μMT mice 21 days after priming with NP-CGG in alum (**C**) and 17 days after NP-CGG boost (**D**) are shown. Lin^-^ was defined as Ter119^−^Mac1^−^Gr1^−^NK1.1^−^CD3^−^CD4^−^CD8^−^

To investigate the immune function of regenerative B cells, we inoculated iB-μMT mice with T cell-dependent antigen (TD Ag) to test the humoral immune response. We immunized iB-μMT mice with 4-hydroxy-3-nitrophenylacetyl-chicken-gamma-globulin conjugates (NP-CGG) and detected the levels of NP-specific IgM and IgG1 antibodies in the sera from immunized mice (Fig. 5A). The iB-μMT mice showed elevated NP-specific IgM, total NP-specific IgG1, and high-affinity NP-specific IgG1 levels compared with μMT mice after the primary immune response (Fig. 5B). After boosting with NP-CGG, increased amounts of total and high-affinity NP-specific IgG1 antibodies were produced quickly from iB-μMT mice, while antibodies were not detected in μMT mice (Fig. 5C). We next assessed the normal formation of germinal center (GC) B cells, memory B cells, and plasma cells, on which adaptive humoral immune protection relies heavily, in iB-μMT mice. Two weeks after NP-CGG immunization, there was robust emergence of plasma cells (B220^low/−^CD138^+^) and NP-specific GC B cells (NP^+^GL7^+^CD38^−^) in the spleens of iB-μMT mice, which was comparable to the B6 mouse counterparts (Fig. 6A). In addition, we detected antigen-specific class-switched IgG1^+^ memory B cells in the spleens of iB-μMT mice on Day 21 after NP-CGG immunization (Fig. 6B), suggesting successful immunoglobulin class switching. Abundant long-lived plasma cells (Lin^−^IgM^−^CD22^−^CD19^−^MHCII^−^CD138^+^) in the bone marrow of iB-μMT mice 3 weeks after NP-CGG immunization were detected, which was comparable to that seen in the B6 mouse counterparts (Fig. 6C). Importantly, long-lived plasma cells could still be detected in the bone marrow of iB-μMT mice at Day 17 after the boost (Fig. 6D). Thus, these results indicate that the regenerative iB cells in the iB-μMT mice produce a primary response and memory response and sustain long-term humoral immune protection, suggestive of a typical adaptive immune response.

## Discussion

In this study, we demonstrated that forced expression of three transcription factors, *Lhx2*, *Hoxa9*, and *Runx1*, can guide B lineage fate commitment and in vivo B lymphopoiesis in B cell-deficient animals. Of note, the results of in vivo lymphopoiesis include pro/pre-B progenitors, immature B cells, and all subsets of mature B-1a, B-1b, FO B, and MZ B cells. We could not detect GFP^+^ HSCs in the bone marrow of iB-μMT mice, and the rare GFP^+^ hematopoietic progenitors isolated from primary recipients could not contribute in secondary recipients (data not shown), indicating that the B lymphogenic potential is determined by *Lhx2*, *Hoxa9*, and *Runx1* at the putative precursor cell stage independent of HSC formation. This HSC-independent approach regenerates a complete humoral system that rescues B cell-related immune responses in animals with inherited B cell deficiency.

ESCs cultured on OP9-DL1 cells preferentially commit to T cells in vitro [65]. However, we only generated robust and transplantable early hematopoietic progenitors using OP9-DL1 cell in vitro, and subsequent B cell development was achieved in vivo via iHPC transplantation. The differentiation of ES cells has been previously performed on OP9 cells to give rise to B cells in vitro; [10, 13, 65] however, B cell development in vitro has limitations including incomplete B cell subsets and defects of functionality. Although transplantation of B220^+^CD93^+^ pro/pre-B cells differentiated from mouse ESCs reconstituted B-1 and B-2 cells in recipients [12], the B cell regeneration was transient and the serum contained extremely low IgM antibodies, which were barely detected 6–8 weeks after transplantation. We noticed that in our system, the iHPCs in the bone marrow microenvironment differentiated into pro/pre-B cells and further matured into entire B cell subsets in vivo, which might avoid the partial failure of modeling the microenvironment of B cell development in vitro. We still detected the presence of induced mature B-1 and B-2 cells and serum antibodies in iB-μMT mice 40 weeks after transplantation.

Constitutive expression of *Lhx2* in natural hematopoietic progenitor/stem cells in vivo led to a myeloproliferative disorder and caused acute leukemia [66], which implies that the iHPCs generated in our study are different from these cells, as our iHPCs did not cause myeloid proliferation. Certain pre-B tumor cell lines expressed *Lhx2* [67], but we did not observe B cell tumors in iB-μMT mice, indicating that simultaneous expression of *Lhx2* with *Hoxa9* and *Runx1* starting from an early stage of hematopoietic development prior to definitive HSC occurrence leads to no obvious tumorigenic effect. In addition, single-cell RNA-seq showed typical expression patterns of surface markers, transcription factors and essential regulators, and pre-BCR complexes in induced pro-B and large pre-B cells, which suggests normal early B lymphopoiesis in iB-μMT mice. Of note, *Ikzf1*, *Spi1*, *Tcf3*, and *Pax5* are normally expressed in induced B cell progenitors, which ensure tumor-free lymphopoiesis as reduction or loss of any of these master factors is associated with B cell leukemia [68–71].

Synergistic expression of *Runx1* and *Hoxa9* during mESC differentiation resulted in an iHPC population that preferentially contributed to T lymphopoiesis in vivo [22]. In this study, using the same induction protocol, coordination of *Lhx2*, *Hoxa9*, and *Runx1* promoted B lymphopoiesis instead of T lymphopoiesis in vivo. It has been reported that T cell development is blocked by the expression of *Lhx2* in vivo in HSPCs [72]. Surprisingly, synergistic expression of the *Lhx2* and *Runx1* transcription factors preferentially determined T cell lineage fate using the same induction system (Fig. S6), despite having low efficiency compared with induction using *Runx1* and *Hoxa9*. Thus, our data demonstrate that the synergistic effects of *Lhx2*, *Hoxa9*, and *Runx1* transcription factors are more complicated than simple addition–subtraction effects. It is worth of further investigation to comprehensively understand the epigenetic landscape induced by *Lhx2*, *Hoxa9*, and *Runx1* during hematopoietic fate commitment and subsequent B lymphopoiesis.

In conclusion, this study establishes a novel approach for reconstituting complete B lymphopoiesis in vivo based on a two-step approach of in vitro HPC commitment from PSCs and in vivo lymphopoiesis. The regenerative B cells possess abundant BCR repertoires capable of recognizing numerous different antigens and can restore the adaptive humoral immune response and form immune memory in B cell-deficient mice. This robust induction system of B cell regeneration provides a new tool for the basic study of B cell development and B cell disease modeling. Given that PSCs are not a limited cell resource and can be subjected to gene editing, our study provides insights into the therapeutic applications of regenerative B cells for individuals suffering from inherited B cell defects.

## Supplementary information

Supplementary Figure Legends

Supplementary Figure 1

Supplementary Figure 2

Supplementary Figure 3

Supplementary Figure 4

Supplementary Figure 5

Supplementary Figure 6

## Data Availability

The BCR sequencing data were deposited in the GEO database under accession number GSE180318, and the scRNA-seq data were deposited in the GEO database under accession number GSE180319. All other data needed to evaluate the conclusions in the paper are present in the paper or the Supplementary Materials.
