# Supplementary material for: Regeneration of immunocompetent B lymphopoiesis from pluripotent stem cells guided by transcription factors
Source: Cell Mol Immunol. 2021 Dec 10;19(4):492–503. doi: 10.1038/s41423-021-00805-6 (PMC8975874; doi:10.1038/s41423-021-00805-6)
Supplement: Supplementary file 1 — Supplementary Figure Legends [file 41423_2021_805_MOESM1_ESM.doc]

**SUPPLEMENTARY FIGURE LEGENDS**

**Fig. S1** **Reconstitution of functional B cells *in vivo* from *iR9X2*-ESC.**

**A** Schematic diagram of establishing *iR9X2*-ESC cell line. A *CAG Pr-GFP-PGK Pr-PuroR* cassette was inserted into the *Hipp11* locus of mouse ESC by homologous recombination to form GFP-ESC clones which were selected by puromycine (1 μg/ mL). Next, a *CAG Pr-rtTA-3×Stop-TRE-Runx1-p2a-Hoxa9-t2a-Lhx2-pA-PGK Pr-HygroR* cassette was inserted into the *Rosa26* locus of GFP-ESC by homologous recombination to form *iR9X2*-ESC clones which were selected by hygromycin B (150 μg/ml). **B** Q-PCR analysis of the expression of *Runx1*, *Hoxa9*, and *Lhx2* in *iR9X2*-ESC after doxycycline induction. **C** *iR9X2*-ESC-derived GFP^+^CD45^+^Mac1^+^ myeloid cells and GFP^+^CD45^+^CD19^+^ B cells in the PB of μMT mice were analyzed by flow cytometry 4 weeks and 8 weeks after transplantation. Five million iHECs-derived hematopoietic progenitors were transplanted into each sublethally irradiated μMT mouse. The mice were fed with water containing doxycycline (1 mg/mL) to induce the generation of B lymphocytes. Two representative mice from five independent experiments are shown. **D** *iR9X2*-ESC-derived B (iB) cells in the PB of C57BL/6 mice were analyzed by flow cytometry. Five million iHECs-derived hematopoietic progenitors were transplanted into each sublethally irradiated (6.5 Gy) C57BL/6 mouse. The mice were fed with water containing doxycycline (1 mg/mL) to induce the generation of B lymphocytes. iHPCs derived hematopoietic cells (GFP^+^CD45^+^Mac1^+^ myeloid cells, GFP^+^CD45^+^CD3/CD4/CD8^+^ T cells, and GFP^+^CD45^+^CD19^+^ B cells) were analyzed 4 weeks after transplantation. GFP^-^CD45^+^CD19^+^ and GFP^-^CD45^+^CD3/4/8^+^ gates of the same C57BL/6 recipients are shown as control. Data from two representative mice are shown. **E** *iRunx1-p2a-Hoxa9-t2a-Lhx2-ESC*-derived B (iB) cells in the PB of *Rag1*^-/-^ mice were analyzed by flow cytometry. iHPCs were induced from GFP negative *iRunx1-p2a-Hoxa9-t2a-Lhx2-ESC, and* five million iHPCs (CD45.2^+^) were transplanted into each sublethally irradiated CD45.1^+^ *Rag1*^-/-^ mouse (3.5 Gy). The mice were fed with water containing doxycycline (1 mg/mL). Hematopoietic cells (CD45.2^+^Mac1^+^ myeloid cells, CD45.2^+^CD3 ^+^ T cells, and CD45.2^+^CD19^+^ B cells) in the peripheral blood of *Rag1*^-/-^ recipients were analyzed at week 4 after transplantation. CD45.2^-^CD19^+^ and CD45.2^-^CD3/4/8^+^ gates of the same CD45.1^+^ *Rag1*^-/-^ recipients are shown as control. Data from two representative mice are shown.

**Fig. S2 Long term serum antibodies levels in iB-μMT mice.**

Sera were collected from iB-μMT mice (18 to 40 weeks after transplantation) and μMT mice (n=7 per group). The different isotypes of antibodies (IgM/IgG1/IgG2b/IgG2c/IgG3/IgA) were measured by ELISA. Each symbol represents an individual mouse; small horizontal lines indicate the mean. *P < 0.05, **P < 0.01, and ***P < 0.001 (Independent-sample student t test and Mann-Whitney U test).

**Fig. S3 The regenerative B cell subsets in iB-μMT mice.**

**A, B** *iR9X2*-ESC-derived B (iB) cells in the bone marrow and spleen of μMT mice were analyzed by flow cytometry. Each μMT recipient was transplanted with five million *iR9X2*-iHPCs collected at day 21. GFP^+^CD45^+^Mac1^+^ myeloid cells, GFP^+^CD45^+^CD3/CD4/CD8^+^ T cells, and GFP^+^CD45^+^CD19^+^ B cells were analyzed 2 weeks (**A**), 4 weeks (**B**), and 8 weeks (**B**) after transplantation. GFP^-^CD45^+^CD19^+^ gates of the same *iR9X2*-μMT recipients are shown as control. Data from six representative mice are shown. **C** Flow cytometry analysis of transitional B cells in the spleen of iB-μMT mice. Each recipient was transplanted with five million *iR9X2*-iHPCs collected at day 21. iB-μMT mice were sacrificed and analyzed 2 weeks, 4 weeks, and 8 weeks after transplantation, respectively. Representative FACS plots from three iB-μMT mice are shown. **D** Flow cytometry analysis of B-1 cells in the spleen of iB-μMT mice. Each recipient was transplanted with five million *iR9X2*-iHPCs collected at day 21. iB-μMT mice were sacrificed and analyzed 8 weeks, 12 weeks, and 24 weeks after transplantation, respectively. Representative FACS plots of B-1a (GFP^+^CD45^+^CD19^+^B220^-/low^CD43^+^CD23^-^CD5^+^) and B-1b (GFP^+^CD45^+^CD19^+^B220^-/low^CD43^+^CD23^-^CD5^-^) from three iB-μMT mice are shown. **E, F** All major mature B cell subsets were analyzed by flow cytometry at week 40 after transplantation. Immuno-phenotypic plots of B-1a, B-1b, FO B, and MZ B cells in the spleen of iB-μMT mice (**E**) were depicted. And immune-phenotypic plots of B-1a, B-1b, and B-2 cells in the peritoneal cavity of iB-μMT mice (**F**) were depicted. Data from one representative mouse are shown. **G** Bone marrow cells were isolated from iB-μMT mice 6 weeks after transplantation, and flow cytometry plots of pro/pre-B cells, immature B cells, and mature B cells were depicted. Data from one representative mouse are shown. Lin^-^ was defined as Ter119^-^Mac1^-^Gr1^-^NK1.1^-^CD3^-^CD4^-^CD8^-^.

**Fig. S4 Sorting gates of bone marrow regenerative progenitors and the expression patterns of selected early B progenitors related genes.**

**A** Bone marrow cells were sorted based on GFP^+^CD45^+^Lin^-^ surface markers for single cell sequencing at day7.5 after transplantation, Lin^-^ was defined as CD3^-^CD4^-^CD8^-^Ter119^-^Mac1^-^Gr1^-^NK1.1^-^. **B** Violin plots show the expression profile of VDJ recombination related enzymes genes (*Rag1*, *Rag2*, and *Dntt*), pre-BCR complex related genes (*Vpreb2* and *Cd79b*), and early B cell development related transcription factors (*Spi1*, *Tcf3*, *Foxo1*, *Bcl11a*, and *Pax5*). The expression value of each gene was normalized by ‘LogNormalize’ method and illustrated by VlnPlot of Seurat. **C** The dotplot present the average expression of marker genes. The average expression value were z-score transformed. The color indicates average expression value and size is proportional to the percentage of cell population with non-zero expression value of selected gene. **D** Table of differential expressed genes between pro-B and large pre-B population. Genes with increased or decreased expression in each population were identified by the Seurat FindConservedMarkers function. The direction of expression is relative. Upregulation means gene expression increased in large pre-B population against pro-B population, while downregulation means gene expression decreased in large pre-B population against pro-B population. The avg_logFC was average of fold difference (log-scale) between the two populations, pct.pro-B and pct.large pre-B was the cell fraction with detected non-zero gene expression in pro-B and larger pre-B, respectively. The p_val, was the p value and p_val_adj was adjusted p-value based on bonferroni correction.

**Fig. S5 Projection of induced B progenitors populations onto wildtype B progenitors.**

**A** UMAP visualization of integrated induced B and wildtype B progenitors. Embeddings of pro-B and large pre-B populations in our study (iB) is on the left and embeddings of wildtype mouse CD19^+^ B progenitors (WT-B, 1195 cells, GSE114793) is on the right. The color indicates cell identities. **B** The cell fraction of different predicted identities of induced pro-B (ipro-B) and induced large pre-B (ilarge pre-B) populations using the pro-B, small pre-B, and large pre-B populations of GSE114793 as reference. Seurat package were implemented for projecting cells in our study onto the wildtype cell-types.

**Fig. S6 Flow cytometry analysis of hematopoietic cells derived from *iRunx1-Lhx2*-ESC in the peripheral blood (PB) of *Rag1*^-/-^ mice.**

*iRunx1-Lhx2*-ESC-derived iHECs were co-cultured with OP9-DL1 stromal cells for 15 days to obtain enough iHPCs, then 3 million iHPCs were transplanted into each sublethally irradiated *Rag1*^-/-^ mouse (3.5 Gy). The mice were fed with water containing doxycycline (1 mg/mL). Hematopoietic cells (Mac1^+^ myeloid cells, CD3/CD4/CD8^+^ T cells, and CD19^+^ B cells) in the peripheral blood of *Rag1*^-/-^ recipients were analyzed by flow cytometry at week 5 and week 8 after transplantation. Data from two representative mice are shown.
