# Supplementary figures and images for: Regeneration of immunocompetent B lymphopoiesis from pluripotent stem cells guided by transcription factors
Source: Cell Mol Immunol. 2021 Dec 10;19(4):492–503. doi: 10.1038/s41423-021-00805-6 (PMC8975874; doi:10.1038/s41423-021-00805-6)

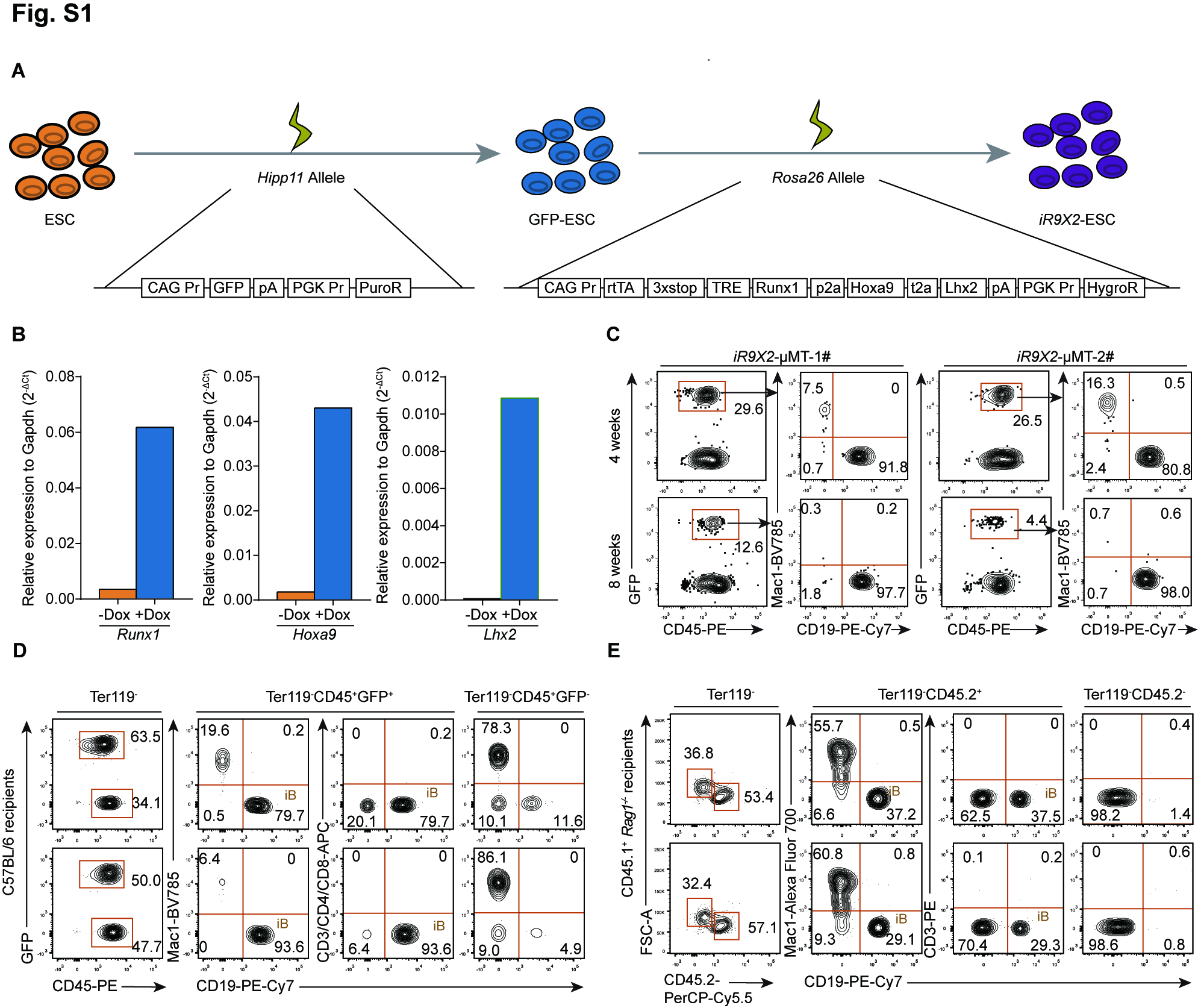

Supplement: Supplementary file 2 — Supplementary Figure 1 [file 41423_2021_805_MOESM2_ESM.tif]

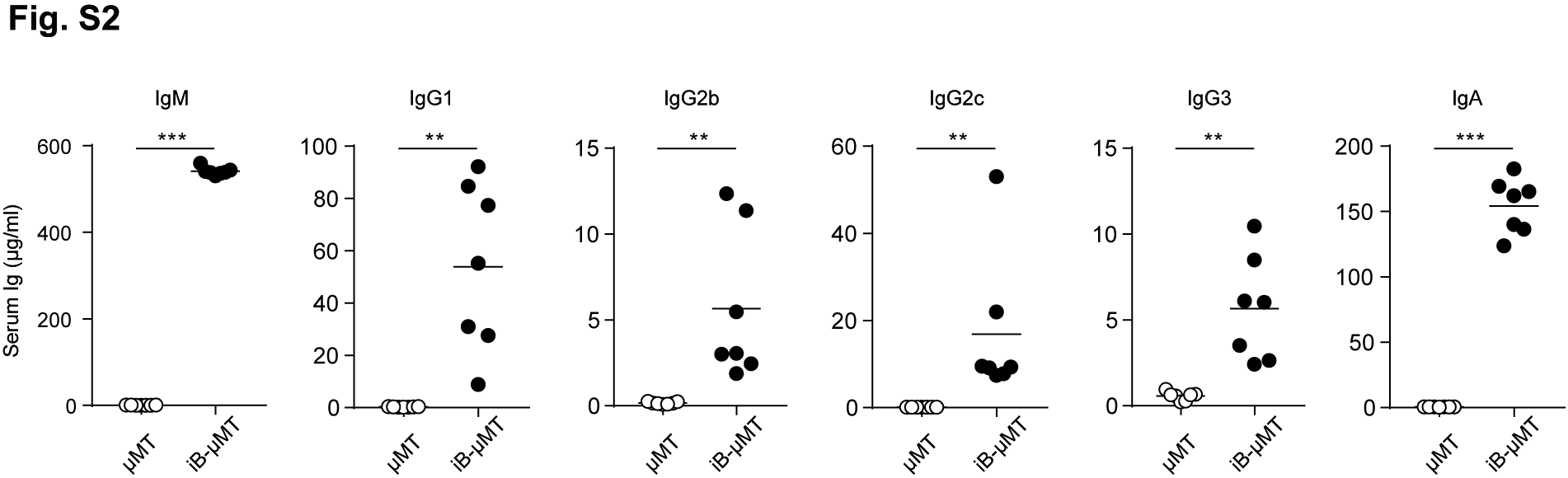

Supplement: Supplementary file 3 — Supplementary Figure 2 [file 41423_2021_805_MOESM3_ESM.tif]

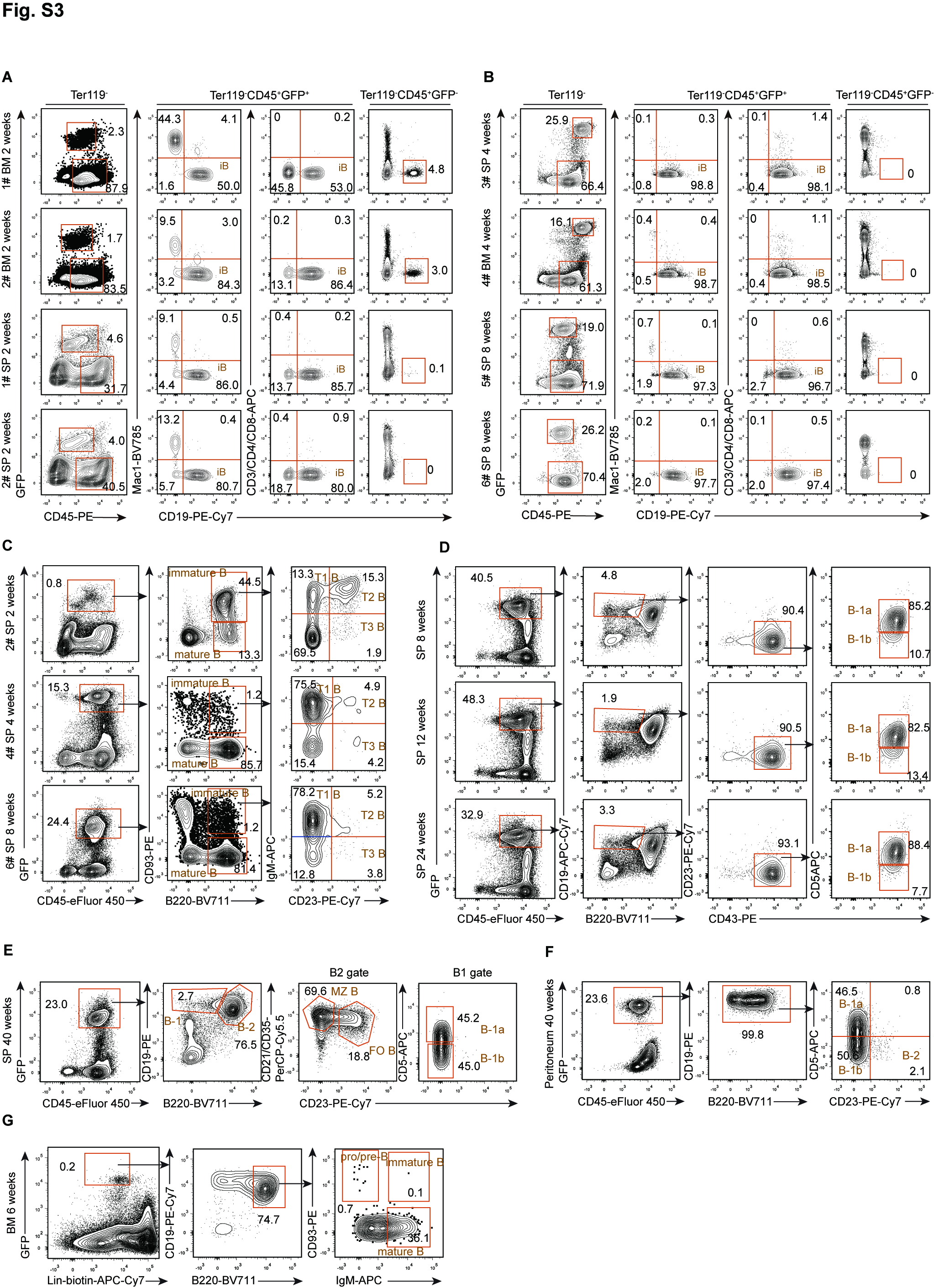

Supplement: Supplementary file 4 — Supplementary Figure 3 [file 41423_2021_805_MOESM4_ESM.tif]

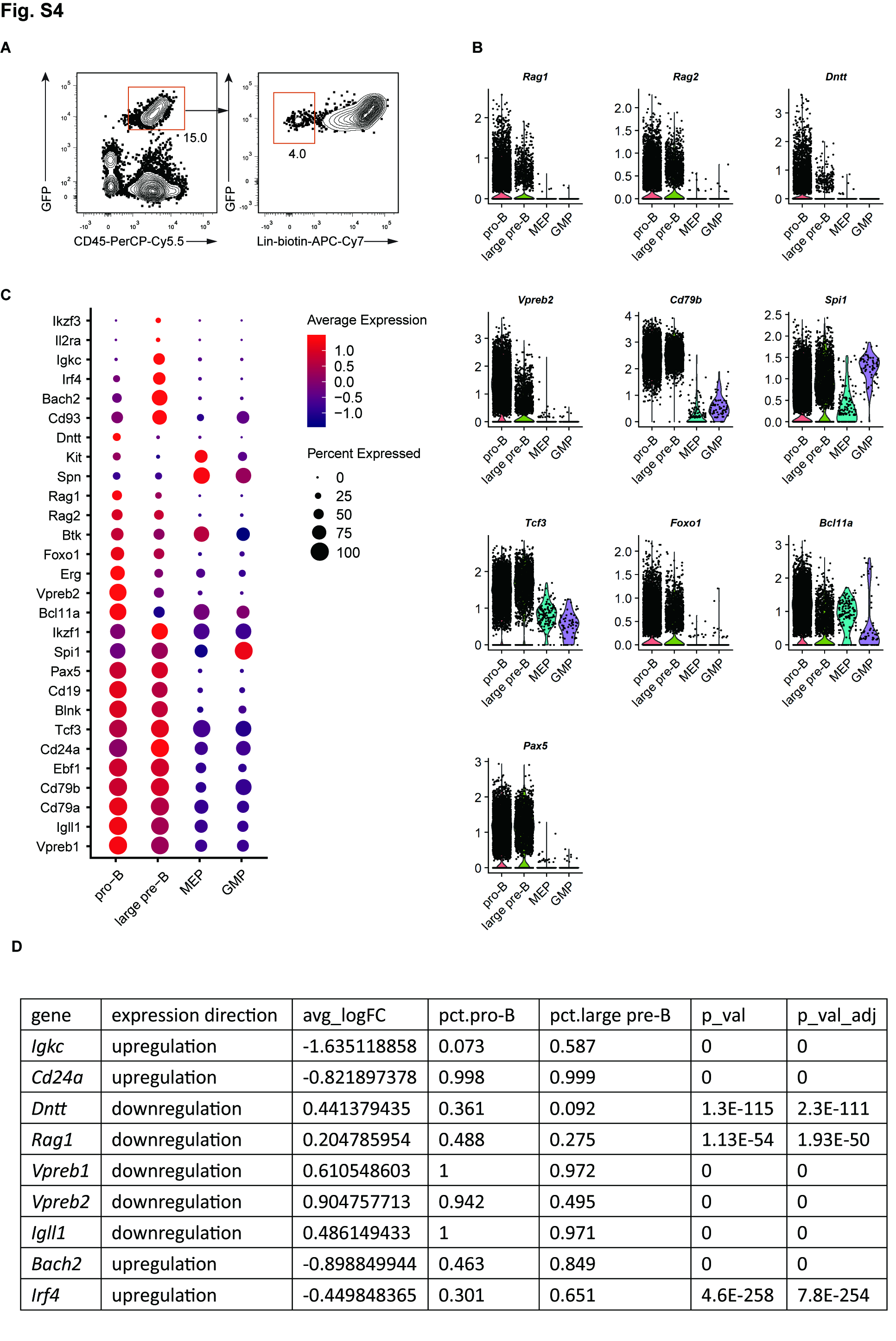

Supplement: Supplementary file 5 — Supplementary Figure 4 [file 41423_2021_805_MOESM5_ESM.tif]

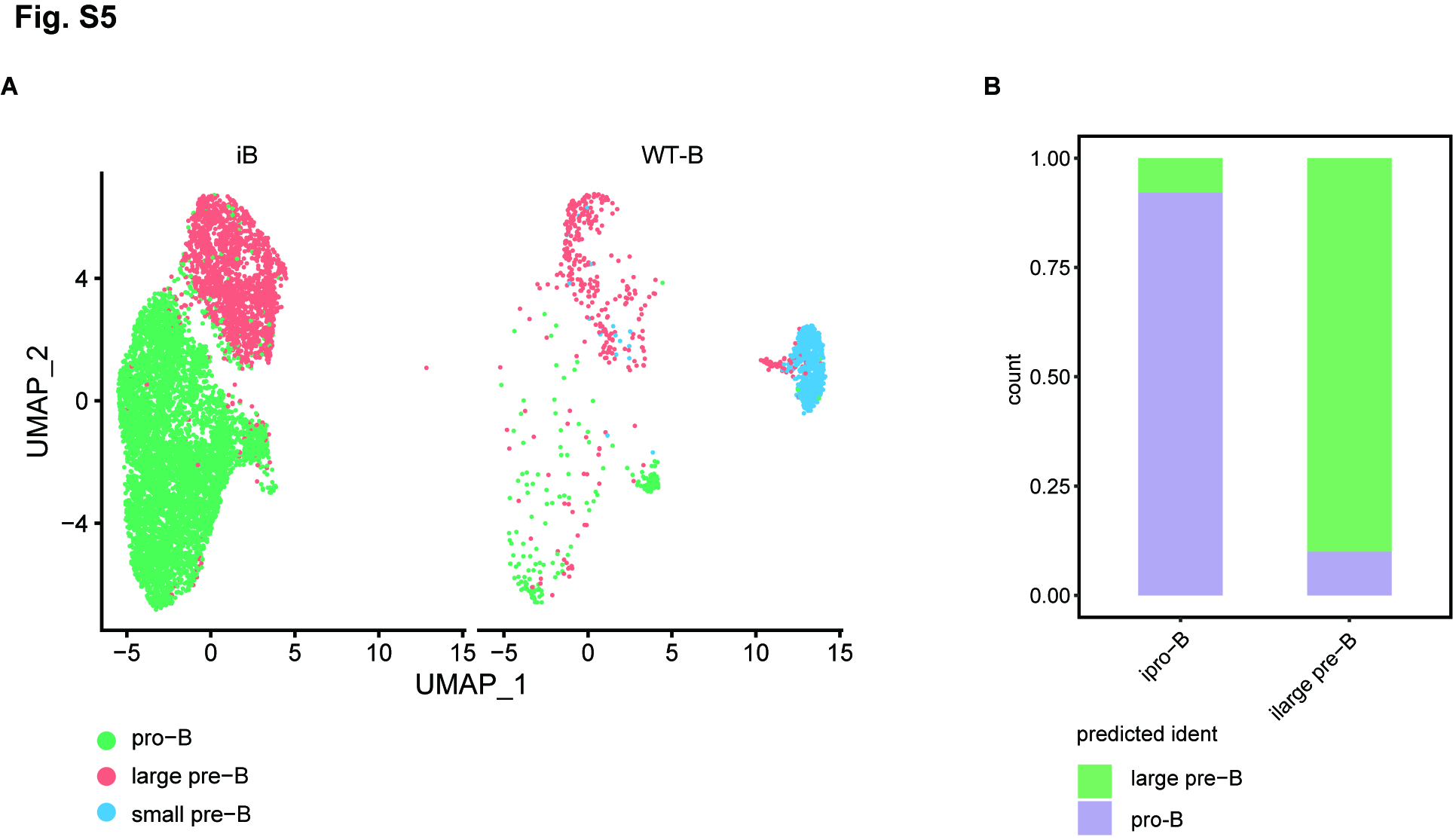

Supplement: Supplementary file 6 — Supplementary Figure 5 [file 41423_2021_805_MOESM6_ESM.tif]

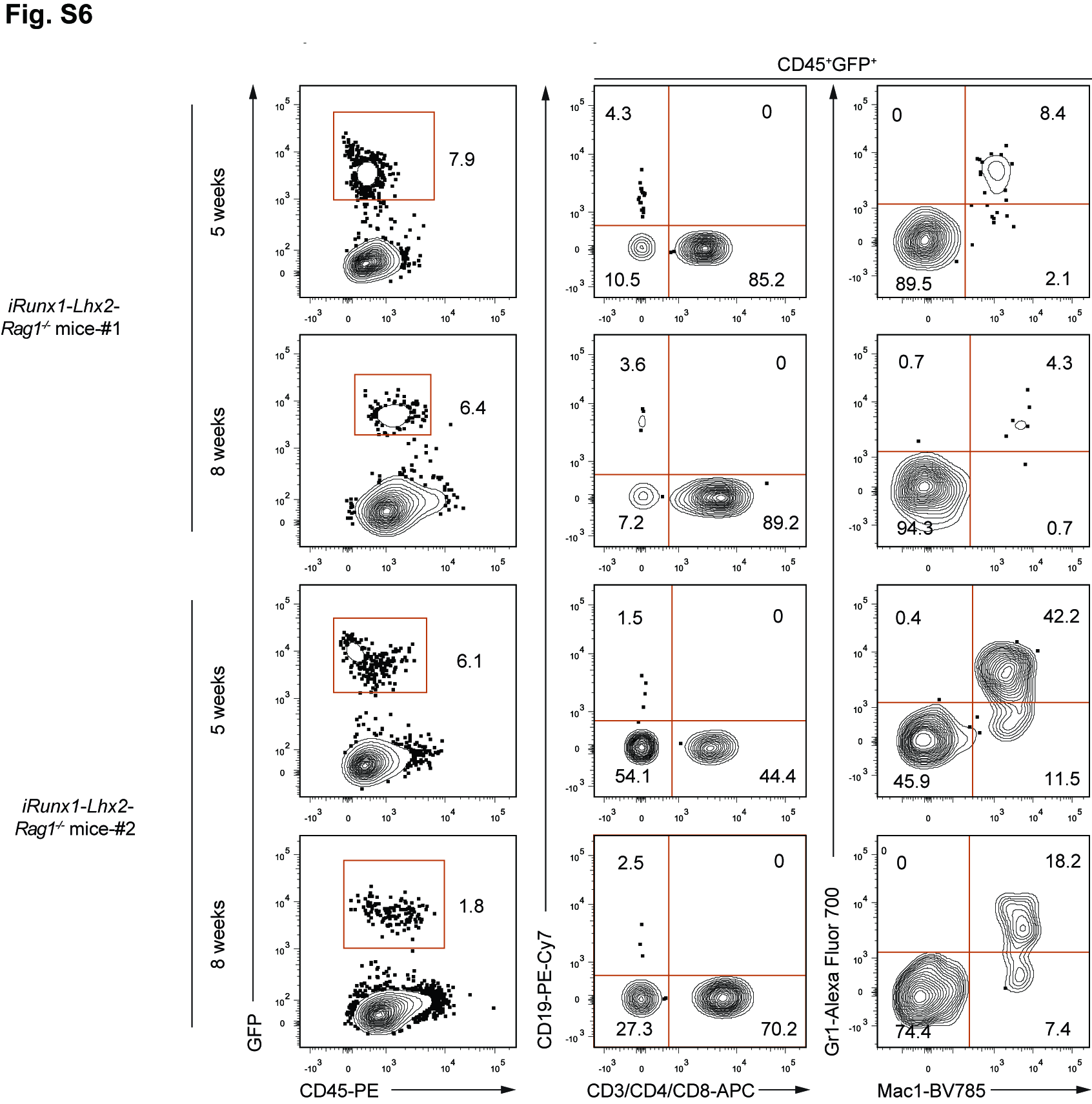

Supplement: Supplementary file 7 — Supplementary Figure 6 [file 41423_2021_805_MOESM7_ESM.tif]
